# Brain Expressed microRNAs Implicated in Schizophrenia Etiology

**DOI:** 10.1371/journal.pone.0000873

**Published:** 2007-09-12

**Authors:** Thomas Hansen, Line Olsen, Morten Lindow, Klaus D. Jakobsen, Henrik Ullum, Erik Jonsson, Ole A. Andreassen, Srdjan Djurovic, Ingrid Melle, Ingrid Agartz, Håkan Hall, Sally Timm, August G. Wang, Thomas Werge

**Affiliations:** 1 Research Institute of Biological Psychiatry, Sct. Hans Hospital, Roskilde, Denmark; 2 Bioinformatics Centre, Institute of Molecular Biology, University of Copenhagen, Copenhagen, Denmark; 3 Department of Clinical Immunology, Rigshospitalet, Copenhagen, Denmark; 4 Human Brain Informatics, Department of Clinical Neuroscience, Psychiatry Section, Karolinska Institutet and Hospital, Stockholm, Sweden; 5 TOP-project, Department of Psychiatry, Ullevål University Hospital, Oslo, Norway; 6 University Department of Psychiatry, Psychiatric Centre Frederiksberg, Frederiksberg, Denmark; 7 University Department of Psychiatry, Psychiatric Centre Amager, Copenhagen, Denmark; 8 Centre for Pharmacogenomics, University of Copenhagen, Copenhagen, Denmark; Innsbruck Medical University, Austria

## Abstract

**Background:**

Protein encoding genes have long been the major targets for research in schizophrenia genetics. However, with the identification of regulatory microRNAs (miRNAs) as important in brain development and function, miRNAs genes have emerged as candidates for schizophrenia-associated genetic factors. Indeed, the growing understanding of the regulatory properties and pleiotropic effects that miRNA have on molecular and cellular mechanisms, suggests that alterations in the interactions between miRNAs and their mRNA targets may contribute to phenotypic variation.

**Methodology/Principal Findings:**

We have studied the association between schizophrenia and genetic variants of miRNA genes associated with brain-expression using a case-control study design on three Scandinavian samples. Eighteen known SNPs within or near brain-expressed miRNAs in three samples (Danish, Swedish and Norwegian: 420/163/257 schizophrenia patients and 1006/177/293 control subjects), were analyzed. Subsequently, joint analysis of the three samples was performed on SNPs showing marginal association. Two SNPs rs17578796 and rs1700 in hsa-mir-206 (mir-206) and hsa-mit-198 (mir-198) showed nominal significant allelic association to schizophrenia in the Danish and Norwegian sample respectively (P = 0.0021 & p = 0.038), of which only rs17578796 was significant in the joint sample. *In-silico* analysis revealed that 8 of the 15 genes predicted to be regulated by both mir-206 and mir-198, are transcriptional targets or interaction partners of the JUN, ATF2 and TAF1 connected in a tight network. JUN and two of the miRNA targets (CCND2 and PTPN1) in the network have previously been associated with schizophrenia.

**Conclusions/Significance:**

We found nominal association between brain-expressed miRNAs and schizophrenia for rs17578796 and rs1700 located in mir-206 and mir-198 respectively. These two miRNAs have a surprising large number (15) of targets in common, eight of which are also connected by the same transcription factors.

## Introduction

Schizophrenia is a severe psychiatric disorder with a strong and complex genetic disposition [1], but still with a largely unknown etiology. Most research in schizophrenia genetics has been focused on protein encoding genes. However, the advent of tiling arrays has shown that a much larger fraction of the human genome is actively transcribed [2]–[4]. Among the transcripts a growing number of genes that encode active RNA species have been identified, suggesting that regulatory RNAs also need to be considered when trying to elucidate the etiology of diseases[5].

microRNAs (miRNA) constitute one class of the newly discovered non-coding RNAs. The primary miRNA transcripts are characterized by a hairpin structure, which guides the subsequent processing of the miRNA into the mature form of a short (19–24 nt) single stranded molecule. miRNAs have been shown to control mRNA stability and translation by binding to complementary sequence motives in the target mRNAs[6]. Individual miRNAs have been shown experimentally to affect the mRNA levels of dozens of genes [7], thus supporting *in silico* analyses [7], [8] and suggesting that individual miRNA have pleiotrophic effects on cellular processes.

A growing number of miRNAs are being experimentally cloned from human tissues and a relatively large number of these miRNAs are expressed in the brain [8], [9]. The presence of miRNAs in adult human brain tissues suggests that they are involved in maintaining brain function [10]. At present, little is known about the functional mechanisms, which are regulated by brain-expressed miRNAs but predictions from computational algorithms suggest that genes involved in “neurogenesis” are over-represented among miRNA target genes [11]. Indeed, *in vitro* studies have shown that miRNAs affects neuronal differentiation [12], [13] and identity [14]. Moreover, the studies, which have been conducted so far, provide evidence that some miRNAs are localized to the synaptosomal complex [15], [16] and in depth characterization has shown that mir-134 modulate synapse plasticity in rat hippocampus by repressing spine formation [17]. In addition, two miRNAs (lsy-6 and mir-273) have been implicated in neuronal pattering during the formation of left-right asymmetry of two morphologically bilateral taste receptor neurons in *Caenorhabditis elegans* [18], [19].

Due to their regulatory properties, their presumed pleiotropic effects and their abundance in the brain, it has been suggested that non-coding RNAs could be involved in the pathogenesis of schizophrenia [5], [20]. Some of the most consistent pathological findings in schizophrenia includes abnormal cyto-architecture [21], left-right asymmetry of brain hemispheres [22], [23] as well as reductions in dendritic spine density and the number of synapses [24], [25]. With the current knowledge that miRNAs are involved in these processes and that alterations in the interactions between miRNAs and their targets may form a potent source of phenotypic variation, brain-expressed miRNAs qualify as candidate genes for schizophrenia. A novel report on differential gene expression of 16 miRNAs in the prefrontal cortex in schizophrenia supports this idea [26]. Therefore, we have investigated if genetic variants within brain-expressed miRNAs are associated with schizophrenia using a case-control study design.

## Methods

### Identification of brain-expressed miRNAs from the literature

A systematic search in PUBMED (release June 14. 2006) for papers on brain-expressed miRNAs resulted in the identification of 14 references, which include data from microarray and Northern Blotting studies. A total of 101 unique miRNA transcripts have been detected in one or more regions of the human brain. SNPs within +/−100 bp of the mature miRNA transcript were identified from the human genome (NCBI build 36.1).

### Genotyping

Genomic DNA was extracted from whole blood samples and the samples were genotyped using the GoldenGate assay (Illumina Inc) [27] on custom designed Illumina Bead Arrays at the genotyping facility at Uppsala University, Sweden. For this platform, assay performance puts restrictions on the SNP panel that can be tested. Assay performance is calculated as an estimated genotype success rate for each SNP, which accounts for both SNP validation status and assay design for the array. SNPs less than 60 bp apart can not both be genotyped; when this occurred we selected the SNP within the sequence coding for the mature miRNA, followed by SNPs with the highest estimated success rate and finally SNPs with the highest minor allele frequency (MAF). Based on these criteria it was possible to genotype 31 SNPs within – or in the vicinity of 28 microRNA genes.

### Samples

This study included three independent population-based case-control samples from Denmark, Sweden and Norway. The sample characteristics of the three samples are summarized in table 1.

**Table 1:** Sample characteristics.

	Schizophrenia		Control	
Sample	Women (SD)	Men (SD)	Women (SD)	Men (SD)
Denmark				
N	179	241	418	588
Mean age	45.1 (12.5)	43.1 (12.0)	44.4 (12.2)	42.4 (11.3)
MAFA	27.0 (11.3)	25.7 (8.9)		
Sweden				
N	97	160	111	182
Mean age	57.1 (16.7)	52.3 (13.8)	50.7 (10.1)	50.1 (10.0)
MAFA	25.9 (8.0)	23.8 (5.9)		
Norway				
N	88	75	98	79
Mean age	38.8 (11.5)	35.6 (9.8)	38.0 (10.2)	39.6 (10.2)
MAFA	27.3 (9.6)	26.8 (9.0)		

Mean age is calculated as the expected mean age in 2006; MAFA: mean age at first admission.

#### The Danish sample

The Danish sample included 420 patients who have been recruited to Danish Psychiatric Biobank from the psychiatric departments at the six hospitals in the Copenhagen region. All patients had been clinically diagnosed with schizophrenia according to ICD-10; F20 and F25 without ever having received a diagnosis of mania or bipolar illness (F30-31). An experienced research- and consultant psychiatrist verified high reliability of the clinical diagnoses [28], [29] using OPCRIT semi-structured interviews. The vast majority of the patients (96%) who fulfilled the ICD-10 criteria of schizophrenia also complied with the corresponding DSM-IV standards. At the time of the last assessment the patients had a mean age of 43.7 (+/−12.0) years, a mean age at onset of illness of 27.7 (+/−9.2) years, a mean duration of illness of 16.5 (+/−10.3) and a mean duration of hospitalization of 4.2 (+/−4.5) years. None of these variables differed significantly between men and women. The majority (85%) of the patients were ethnical Danish, i.e. the patients and both parents were born in Denmark, while in a minor fraction of the cases (15%) one parent was Caucasian and born outside Denmark in another North-western European country, primarily in Sweden or Norway, secondarily in Germany, the Netherlands, England or France.

The healthy controls subjects were recruited among 15,000 blood-donors from the Danish Blood Donor Corps in the Copenhagen area. The Donor corps includes >5% of the Danish population who donate blood on a voluntary and unpaid basis. Apparent behavioral abnormality was an exclusion criterion and all individuals stated that they felt completely healthy with a possibility to discuss any health related issues with a physician. Two unrelated healthy control subjects of Danish Caucasian origin were matched to each patient on gender, year of birth and month of birth, with matching ethicality.

The Danish Scientific Committees and the Danish Data Protection Agency approved the study and all the patients have give written informed consent prior to inclusion into the project.

#### The Swedish sample

The Swedish sample included 257 patients who have been recruited from psychiatric clinics in northwestern Stockholm County. All patients had been clinically diagnosed with schizophrenia (n = 226), schizophreniform disorder (n = 8) or schizoaffective disorder (n = 25) according to DSM-III-R/DSM-IV diagnostic criteria based on interviews and record reviews as previously described [30]–[32]. At the time of the last assessment the patients had a mean age of 43.6 (+/−14.2) years, a mean age at onset of illness of 24.6 (+/−6.8) years, and a mean duration of illness of 19.0 (+/−13.8) years. Psychotic women were older when entering the study (46.9+/−15.5 years), and tended to have a higher age at onset (25.8+/−8.0 years) and a longer duration of illness (21.0+/−14.7 years) than men with psychosis (41.6+/−13.1, 23.9+/−5.9 and 17.7+/−13.2 years; Wilcoxon/Kruskal Wallis tests p = 0.007, p = 0.07, and p = 0.09, respectively). All patients were Caucasian. Based on the birth country of the grandparents or greater grandparents, 79%, 12% and 9% of the patients were estimated to be of Swedish, Finnish or other European origin, respectively.

The healthy control subjects were recruited among subjects previous participating in biological psychiatric research at the Karolinska Institute or drawn from a representative register of the population in Stockholm County and interviewed as previously described [31]. All controls were Caucasian and 86%, 6%, and 8% were estimated to be of Swedish, Finnish or other European origin, respectively. The mean age was 40.5 (+/−9.8) years when entering the study. None of the controls suffered from schizophrenia.

The Ethical Committee of the Karolinska Hospital, the Stockholm Regional Ethical Committee and the Swedish Data Inspection Board approved the study. All subjects participated after giving informed consent.

#### The Norwegian sample

The Norwegian sample included 163 patients who had been recruited to the TOP study from all the psychiatric hospitals in the Oslo area. The patients had been diagnosed according to Structural Clinical Interview for DSM-IV (SCID) as schizophrenia (n = 124) schizoaffective (n = 31) and schizophreniform (n = 8). Two clinical professors continuously trained and supervised a group of research fellows in order to secure the quality of the clinical assessments. Reliability of the diagnosis has recently been tested, and the percentage of agreement was 82%, and Kappa 0.77 (95% CI: 0,60–0,94).

At the time of the last assessment the patients had a mean age of 37.1 (+/−10.7) years, a mean age at onset of illness of 27.5 (+/−8.6) years (onset defined as age of first contact with psychiatric health service), and a mean duration of illness of 10.6 (+/−10.5) years. None of these variables differed significantly between men and women. The majority (90%) of the patients were ethnical Norwegian, i.e. the patient and both parents were born in Norway, while in a minor fraction of the cases (10%) one parent was born outside Norway in another North-western European country.

The healthy control subjects were randomly selected from statistical records of persons from the same catchments area as the patient groups. Only subjects born in Norway were contacted by letter and invited to participate. All controls were of Caucasian origin; around 85% had two Norwegian parents, the rest one parent from other European origin. Moreover, all participants had to have Norwegian as their first language or have received their compulsory schooling in Norway. The mean age of the control subjects in 2006 was 39.0 (+/−10.2). The control subjects were screened by interview and with the Primary Care Evaluation of Mental Disorders (PRIME-MD). None of the control subjects had a history of moderate/severe head injury, neurological disorder, mental retardation or an age outside the age range of 18–60 years. Healthy subjects were excluded if they or any of their close relatives had a lifetime history of a severe psychiatric disorder (schizophrenia, bipolar disorder and major depression), a history of medical problems thought to interfere with brain function (hypothyroidism, uncontrolled hypertension and diabetes), or significant illicit drug use.

The Norwegian Scientific-Ethical Committees and the Norwegian Data Protection Agency approved the study and all patients have given written informed consent prior to inclusion into the project.

### Statistics

We have tested for allelic, genotypic – and in a few cases the haplotype effect in each the of Scandinavian samples, and that of the combined sample for those with p<0.1.

Test for Hardy-Weinberg (HW) proportions was performed by chi-square exact tests implemented in the PLINK (v. 0.99r) [33] on all heterozygous markers in cases and control subjects of each Scandinavian sample. SNPs showing nominal HW disequilibrium (p<0.05) in one or more control groups were removed from the subsequent analyses in the corresponding sample(s). The heterogeneity between the three Scandinavian samples was estimated by calculating the fixation index of the all SNPs in the healthy controls, using Stucture2.2 [34]. In addition, Breslow-Day test was used to evaluate the homogeneity of the odds ratios between the three samples.

Test for allelic association and genotypic effects was calculated for all three Scandinavian samples separately and combined using a standard chi squared test. Subsequently, the Cochran-Mantel-Haenszel test for 2×2×*k* tables was used to perform a combined analysis of all three samples for all markers with p<0.10 in one or more samples.

PLINK (v. 0.99r) [33] was used to perform the Breslow-Day test, allele and genotypes association analysis and the Cochran Mantel-Haenszel test. The odds ratio (w. 95% confidence interval (CI^95^)) was manually calculated for nominal significant SNPs.

Three SNPs were genotyped for the hsa-let-7a-3 gene. LD blocks were first defined from the genotype data using the default setting in HAPLOVIEW [35] in all three sample for both schizophrenic patient and control subjects separately. UNPHASED (v. 3.0.3) [36], [37] was then used to estimate haplotype phases, to test for haplotype main effects and to estimate relative risk of individual haplotypes.

### Network identification

The two miRNAs that that showed nominally significant association in the analyses were further analyzed using TargetScanS[8] to identify their predicted gene targets. Genes that were targeted by both miRNAs were identified. This common target (CoT) list was then analyzed using Biomolecular Object Network Databank (BOND™) to identify all their known protein- or DNA- interaction partners. The resulting interaction network of CoTs and interaction partners was analyzed and visualized using Cytoscape[38] to identify potential network(s).

## Results

We selected 101 miRNA genes with known expression in the human brain, by systematic review of the public miRNA literature. In these we identified 46 SNPs within the mature miRNA sequence and in the adjacent +/−100 bp genomic regions that presumably could interfere with miRNA function (i.e. affecting the promoter activity, splicing of the pre-miRNA etc.) or serve as tags for potential unknown SNPs situated within the coding region.

Of the 46 identified SNPs we were able design assays for 31 SNP loci in 28 miRNA gene regions using the Illumina® chip platform (see Methods). Allele frequencies for these 31 SNPs in the Danish, Swedish and Norwegian samples are listed in table 2 and shown to be similar across the three samples. Ten loci were found to be monomorphic, while two SNPs had very low MAF and were only represented in one of the three control samples (Table 2).

**Table 2:** miRNA minor allele frequencies in three Scandinavian samples, allele counts and related p-values for allelic association.

			MAF -Controls			MAF-Cases			HWP^2^ Controls			Allelic Association^3^					
MicroRNA	SNP	Variant^1^	DK	NO	SE	DK	NO	SE	DK	NO	SE	Total	DK	NO	SE	CMH	BD-test
hsa-let-7a-1	rs12338528	G/(A)	0.0005	0	0	0	0	0	1	1	1	NC	0.52	NA	NA	NC	NA
hsa-let-7a-2	rs629367	A/C	0.13	0.17	0.14	0.15	0.15	0.15	0.58	0.42	0.62	NC	0.21	0.56	0.47	NC	0.52
hsa-let-7a-3	rs7288847	T/C	0.37	0.35	0.38	0.36	0.36	0.36	0.59	0.50	0.62	NC	0.91	0.84	0.52	NC	0.84
hsa-let-7a-3	rs731085	C/G	0.36	0.34	0.37	0.36	0.34	0.36	0.54	1	1	NC	0.76	0.96	0.57	NC	0.82
hsa-let-7a-3	rs738559	G/A	0.06	0.06	0.05	0.06	0.06	0.03	0.08	0.74	1.00	0.36	0.84	0.95	*0.07*	0.48	0.23
hsa-mir-100	rs543412	C/T	0.27	0.32	0.28	0.29	0.30	0.29	0.07	0.86	0.47	NC	0.30	0.70	0.79	NC	0.69
hsa-mir-107	rs10509577	A/G	0.07	0.08	0.09	0.07	0.05	0.09	0.64	1	0.15	0.48	0.92	*0.06*	0.81	0.35	0.27
hsa-mir-107	rs11185776	C/(A)	0	0	0.002	0	0	0	1	1	1	NC	NA	NA	0.35	NC	NA
hsa-mir-128b	rs2305234	G/A	0.14	0.12	0.11	0.13	0.09	0.11	0.18	0.74	1	NC	0.58	0.10	0.98	NC	0.40
hsa-mir-182	rs4467881	A/G	0.42	0.46	0.39	0.43	0.42	0.43	0.75	0.76	0.11	NC	0.85	0.31	0.15	NC	0.23
hsa-mir-184	rs12903401	G/C	0.49	0.52	0.45	0.49	0.15	0.45	0.80	0.65	1	NC	0.68	0.95	0.88	NC	0.99
hsa-mir-198	rs1700	C/T	0.12	0.07	0.09	0.10	0.04	0.11	0.07	0.60	0.25	0.12	0.18	**0.04**	0.13	0.35	**0.02**
hsa-mir-206	rs17578796	C/T	0.004	0.014	0.012	0.016	0.006	0.014	1	1	1	**0.041**	**0.002**	0.30	0.81	*0.07*	**0.04**
hsa-mir-216	rs10865292	A/G	0.07	0.09	0.09	0.08	0.09	0.10	0.22	1	1	NC	0.22	0.84	0.70	NC	0.72
hsa-mir-23b	rs1011784	C/G	0.28	0.24	0.28	0.28	0.28	0.25	0.48	1	0.88	NC	0.89	0.26	0.21	NC	0.24
hsa-mir-26a-1	rs7372209	C/T	0.28	0.36	0.32	0.27	0.30	0.29	0.76	1	0.22	0.25	0.48	*0.08*	0.41	*0.09*	0.49
hsa-mir-27a	rs11671784	G/A	0.01	0.01	0.01	0.01	0.01	0.01	1	1	1	NC	0.30	0.59	0.59	NC	0.45
hsa-mir-32	rs7041716	C/A	0.11	0.10	0.12	0.10	0.11	0.14	0.87	0.69	1	NC	0.43	0.81	0.33	NC	0.44
hsa-mir-335	rs3807348	A/G	0.43	0.47	0.46	0.46	0.46	0.46	**0.04**	0.45	0.15	Ex	Ex	0.70	0.85	Ex	Ex
hsa-mir-9-2	rs2304608	C/A	0.14	0.14	0.15	0.16	0.13	0.12	0.42	0.76	0.82	NC	0.22	0.64	0.15	NC	0.15
hsa-mir-95	rs11939078	C/T	0.32	0.33	0.30	0.32	0.30	0.29	0.51	0.07	0.41	NC	0.90	0.48	0.84	NC	0.79

Monomorphic miRNA SNPs: hsa-let-7a-1 (rs7847425),hsa-let-7a-3 (rs9627620),hsa-mir-100 (rs11821130),hsa-mir-125a (rs12975333),hsa-mir-126 (rs7846876),hsa-mir-191 (rs11706445),hsa-mir-221 (rs7050391),hsa-mir-23b (rs3802448)and hsa-mir-99b (rs8112073). NA: Not available, Ex: Excluded, NC: Not calculated, DK: Denmark, SE: Sweden, NO: Norway, CMH: Cochran-Mantel-Haenszel test for 2×2×K stratified tables and BD: Breslow-Day test of homogeneity of odds ratio. ^1^The major allele is give prior to the minor allele ^2^P-values obtained from chisq exact test for Hardy-Weinberg Proportions. ^3^P-values from chisq test for differences in allele proportions.

The remaining 19 miRNA-loci did not deviate significantly from the expected Hardy-Weinberg proportions, with the exception of rs3807348 that was in disequilibrium in the Danish control group (p = 0.039). This SNP was not analyzed further in the Danish sample (table 2). Further, rs10865292 did deviate significantly from the expected Hardy-Weinberg proportions in the Danish patient sample (data not shown), but the SNP was not significantly associated with disease status (table 2). In addition, we calculated the mean Fixation Index for all informative SNPs (F_st_ = 0.0013), and found no sign of stratification between the three Scandinavian samples.

Among the 18 loci, one SNP (rs17578796) in hsa-mir-206 (mir-206) showed overall significant allelic association with schizophrenia (unadjusted p = 0.041, table 2), with the main contribution coming from the Danish sample (unadjusted p = 0.002). The odds ratios of the combined and the Danish samples suggest that the minor allele increases the susceptibility to schizophrenia (OR = 1.86 (CI^95^ 1.03–3.36) and OR = 3.52 (CI^95^ 1.54–8.08, respectively). Furthermore, one SNP (rs1700) in hsa-mir-198 (mir-198) showed significant association with disease in the Norwegian sample (unadjusted p = 0.038) with the minor allele being protective (OR = 0.48 (CI^95^ 0.24–0.96). The Breslow-Day test for homogeneity of both rs17578796 and rs1700 were significant, indicating that there are heterogeneity of the odds ratios in the three populations, and thus consistent with the observed lack of significant association in the overall sample.

Haplotype analysis was done for the hsa-let-7a-3 gene region where 3 SNPs were genotyped pair wise LD (D′) for these 3 SNPs were above 0.98, and there were no association of hsa-let-7a-3 haplotypes overall or in the three population individually (P>0.2).

Correcting for multiple testing (16 independent tests were performed as three of the 18 examined SNPs in hsa-let-7a3 are in strong LD) using Bonferroni correction yields an adjusted the significance level P = 0.05/16∼0.003, which were obtained only by rs17578796 in the Danish sample.

To elucidate the biological signaling network implicated in schizophrenia in this study, the predicted targets of the two associated miRNAs (rs17578796 and rs1700) were identified using TargetScanS. This analysis revealed 440 genes and 148 genes targeted by mir-206 and mir-198, respectively, of which 15 genes were common targets (CoT) of both miRNAs. The first-degree protein- and DNA-interaction partners of the 15 CoTs were identified using BOND™ in order to construct the signaling networks in which they operate. These networks were analyzed and visualized using Cytoscape.

**Figure 1:**
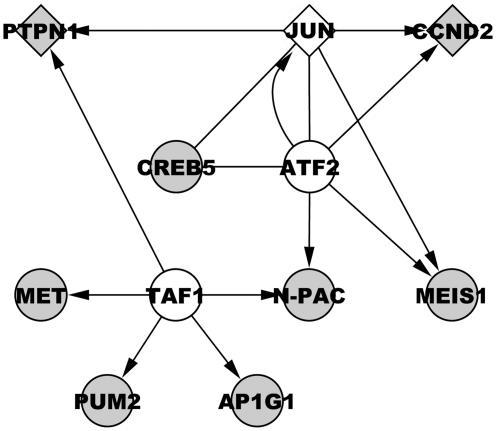
MicroRNA Network. Legend: Circles represent genes with their official HUGO symbols. Grey filled = mir 206/198 targets, white filled = first-degree interaction proteins. Diamond shape = Genes previous found associated with schizophrenia. A Line symbolize an interaction, in which arrow = DNA-protein interaction and no arrow = protein-protein interaction.

Seven networks included only a single CoT and were therefore not analyzed further. One interesting network, however, included eight CoTs connected by three interaction partners (the transcription factors TAF1, JUN and ATF2), as shown in figure 1. Interestingly, two of the genes in this network have previously been associated with schizophrenia as deregulated in postmortem brains (CCND2 [39]) or positioned under a highly significant linkage peak (PTPN1[40]). Also, the three interaction partners are expressed in prefrontal cortex (according to GNF SymAtlas© v1.2.4) that has been implicated in schizophrenia[41]. Finally, JUN is up-regulated in post-mortem brains of schizophrenia patients[42] whereas the neuro-specific expression of TAF1 has been related to another neurological disorder: X-linked dystonia-parkinsonism[43].

## Discussion

To our knowledge this is the first study on genetic variation in miRNA in relation to schizophrenia. The list of known miRNAs is continuously growing and with that the number of miRNAs known to be expressed in the brain. We have analyzed 31 miRNA variants - or approximately three-quarters of the identified polymorphic loci – located in 28 miRNA gene regions or one fourth of the miRNAs expressed in the developing- or adult human brain (28). We find that of the 31 SNPs genotyped, ten were monomorphic in all three Scandinavian samples, two had very low frequency and one deviated from that distribution expected under Hardy-Weinberg proportions in one of the control samples.

Among the remaining 18 SNPs, we find significant allelic association of two SNPs. In mir-198, rs1700 was found associated with schizophrenia only in the Norwegian sample, while the rs17578796 (in mir-206) was disease associated in the total sample and the Danish, the latter being resistant to correction for multiple testing. The two SNPs are positioned in the region flanking the miRNA genes and are therefore unlikely to interfere directly with the biological activity of the miRNAs. Our findings are not consistent across the three samples, illustrated by the heterogeneity of the odds-ratio of both mir-206 and mir-198 (Breslow-Day test, table 2). Though the frequency of rs17578796 resembles that of the reported in Europeans by dbSNP(NCBI), its low frequency highlight the requirement for additional genotyping of independent samples to exclude type I error. Given that the observed associations are indeed true, this heterogeneity merits particular attention.

First, Scandinavians are generally considered to be well suited for genetic (replication-) studies, being ethnically homogeneous populations that only recently have been subject to non-Caucasian immigration. This notion is ensured as all study subjects are born in the Scandinavia and the vast majority has two Scandinavian born parents. A small minority has one Caucasian parent born in North-Western Europe, but outside Scandinavia. However, exclusion of patients with one non-Danish parent from the Danish sample had no effect on the results (data not shown). This is consistent with the recent study by the Welcome Trust Consortium seems to suggest that ethnic admixture is of little concern in genetics studies once non-European immigration is eliminated from the study [44]. This does not exclude, however, that even the limited presence of subjects with partly Finnish ancestry in the Swedish sample may introduce a bias that in this study may have blurred true positive findings. To test the notion of relatively homogeneous Scandinavian populations within the experimental setting of this study, we estimated the mean Fixation Index. Although we found no indication of ethnic admixture (F_st_ = 0.0013), a confounding effect on a single locus cannot be excluded.

Second, the diagnostic reliability of the three samples has been ascertained thoroughly and is unlikely to be the cause of the observed heterogeneity. In contrast, the three samples are different in other clinical aspects that may confound our findings. Most importantly, the Norwegian patients are generally younger with a shorter duration of illness than the considerably more chronic and poor outcome of the Danish and Swedish patients.

Finally, the observed association may arise from the two SNPs being in LD with unknown, causal at-risk alleles in the miRNA genes. These might be located outside – and even quite distant from – the selected gene region that we have targeted in this study, e.g. in the promoter region of the coding (host) genes in which many miRNAs are embedded. Alternatively, the genotyped SNPs may capture a number of very low frequency or ‘private’ SNPs in the miRNAs that will only be discovered by direct sequencing.

By *in-silico* analysis, we find fifteen targets shared by the two miRNAs. This constitutes more than a five-fold over-representation compared to the expected (2.7 common targets (CoTs)) of a random selection of miRNAs targets among the roughly 24,000 known human genes. Furthermore, we find that eight of these 15 CoTs are located in a remarkably simple or compact signaling network with several components having been implicated in schizophrenia or related brain disorders [39]–[43]. This finding lends support to the associations observed in our study, but also suggest that the identified candidate network should be further investigated based on the hypothesis that multiple variants independently or combined may perturb the biological activity of the network.
